# Multiparametric cardiac MRI in the diagnosis of transplant rejection in patients after orthotopic heart transplantation

**DOI:** 10.1007/s00330-026-12631-3

**Published:** 2026-05-26

**Authors:** Justyna Maria Sokolska, Mateusz Sokolski, Jerzy Nożyński, Dominika Konecka-Mrówka, Aleksandra Goraj-Zając, Magdalena Cielecka, Maciej Bochenek, Roman Przybylski, Michał Zakliczyński, Wojciech Kosmala, Robert Manka

**Affiliations:** 1 Division of Cardiovascular Imaging, Institute of Heart Diseases, Faculty of Medicine, Wroclaw Medical University, Wroclaw, Poland; 2 Centre for Heart Diseases, Jan Mikulicz-Radecki University Hospital, Wroclaw, Poland; 3 Clinical Department of Heart Transplantation and Mechanical Circulatory Support, Department of Cardiac Surgery and Heart Transplantation, Institute of Heart Diseases, Faculty of Medicine, Wroclaw Medical University, Wroclaw, Poland; 4 Department of Histology, Faculty of Medical Science in Zabrze, Medical University of Silesia, Silesian Center for Heart Diseases, Zabrze, Poland; 5 Department of Cardiology, University Heart Center, University Hospital Zurich, University of Zurich, Zurich, Switzerland; 6 Diagnostic and Interventional Radiology University Hospital Zurich, Zurich, Switzerland; 7 Institute for Biomedical Engineering, University and ETH Zurich, Zurich, Switzerland

**Keywords:** Cardiac allograft rejection, Graft rejection, Heart transplantation, Magnetic resonance imaging, Tissue characterization

## Abstract

**Objectives:**

Heart transplantation (HTx) remains the main long-term treatment for end-stage heart failure. Due to the high risk of acute cellular rejection (ACR), HTx recipients undergo multiple endomyocardial biopsies to monitor graft tolerance. This prospective, single-center study evaluated quantitative magnetic resonance imaging (MRI), particularly T1- and T2-mapping, as noninvasive tools for rejection monitoring.

**Materials and methods:**

The study included 17 adult HTx recipients (men, 88%; age, 53 ± 13 years) enrolled within 1 month after transplantation and 10 controls without structural heart disease. Htx recipients underwent 5 to 6 serial cardiac MRI scans with T1- and T2-mapping, coinciding with routine endomyocardial biopsies, to correlate imaging findings with histopathological evidence of ACR. Cardiac MRI relaxation times were compared based on the biopsy evidence of ACR.

**Results:**

During the 6-month follow-up, HTx recipients underwent 87 cardiac MRI scans, with 13 ACR episodes reported in 9 patients (53%). They had significantly higher T1- and T2-mapping values than controls. Cardiac biomarkers, NT-proBNP and troponin, did not differ between patients with or without ACR. Global T2-mapping and regional abnormalities, particularly in septal segments, identified rejection, with a cutoff of 51 ms showing high specificity (92%) but modest sensitivity (46%) for ACR. In contrast, T1-mapping values were elevated only in selected myocardial segments, showing a consistent inferoseptal pattern, without differences in global myocardial T1 values between ACR and non-ACR patients, limiting their usefulness for identifying ACR.

**Conclusion:**

Noninvasive cardiac MRI, particularly T2-mapping, may help assess the risk of cardiac graft rejection and complement biopsy-based surveillance in HTx recipients.

**Key Points:**

***Question*** *Monitoring acute cellular rejection (ACR) after heart transplantation is crucial, but whether noninvasive imaging can safely reduce the number of endomyocardial biopsies is still debatable.*

***Findings*** *Multiparametric cardiac magnetic resonance combining T1- and T2-mapping may improve diagnostic assessment and support more targeted use of invasive endomyocardial biopsies.*

***Clinical relevance*** *T2-mapping appears to be a promising noninvasive biomarker for detecting ACR in heart transplant recipients within the 6 months post-surgery, whereas T1-mapping shows regional differences but lacks consistency across the myocardium, having limited diagnostic utility in detecting acute inflammatory changes.*

**Graphical Abstract:**

**Figure:**
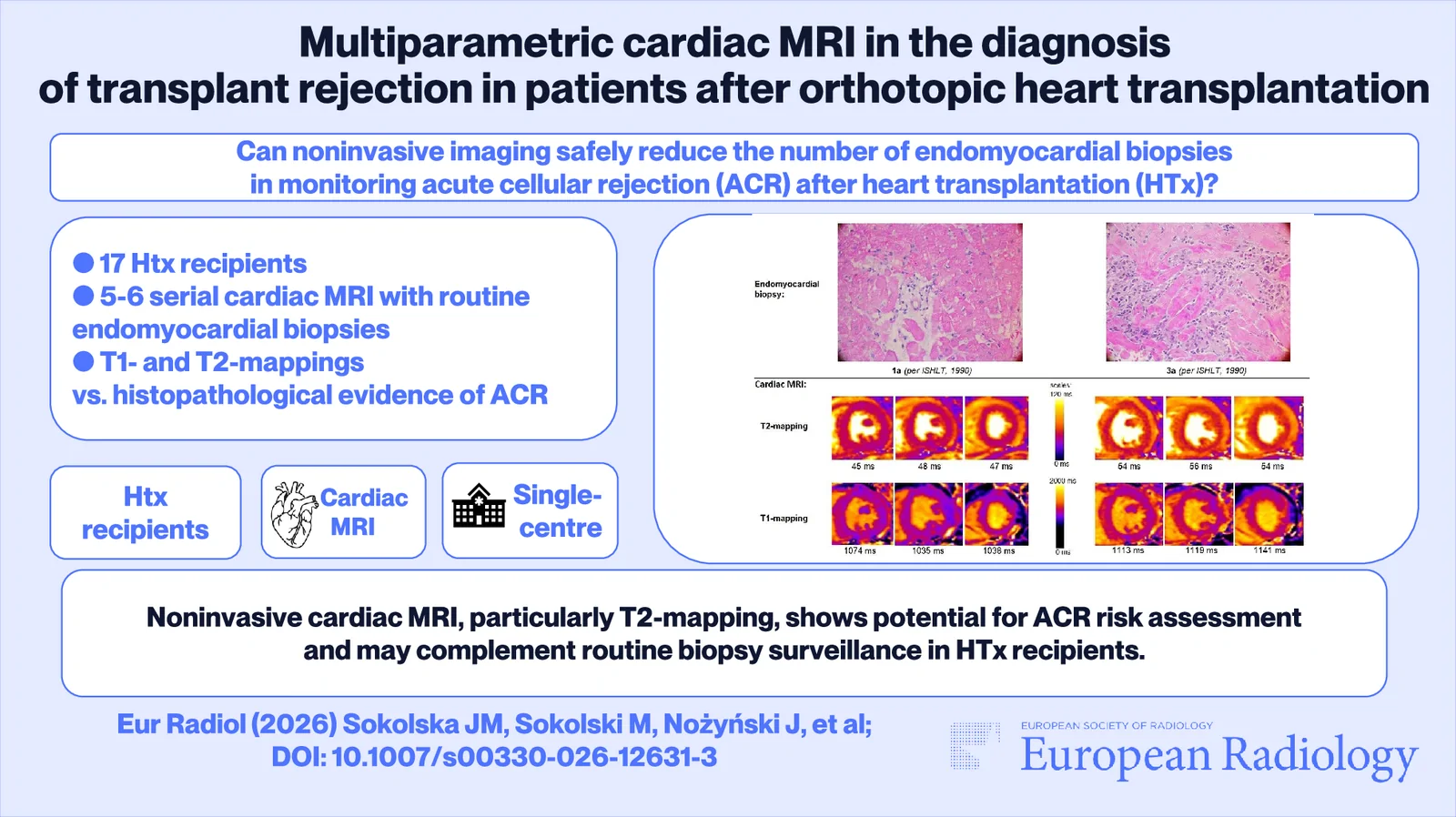

## Introduction

Despite significant progress in medicine, orthotopic heart transplantation (HTx) remains the main long-term treatment option for patients with advanced end-stage heart failure [1, 2]. One of the major challenges following organ transplantation is the risk of donor tissue rejection by the recipient’s immune system, which most often occurs within the first months after surgery [3]. To prevent this potentially fatal complication, transplant recipients require lifelong immunosuppressive therapy [3]. The gold standard for monitoring graft tolerance and rejection in HTx recipients is invasive endomyocardial biopsy [1–3]. Myocardial biopsy samples are subjected to histopathological evaluation and classified according to the 4-grade scale of the International Society of Heart-Lung Transplantation (ISHLT) [4].

Complications of endomyocardial biopsy, including right ventricular perforation, cardiac tamponade, tricuspid valve damage, pulmonary embolism, infection, bleeding, and right ventricular fistula, occur in approximately 1–2% of cases [5–7]. Because of its invasiveness, the need for frequent repetition, and potential complications, there is an ongoing search for alternative noninvasive diagnostic techniques that could provide comparable clinical information with improved patient safety [8–12]. One such technique may be cardiac magnetic resonance imaging (MRI) with quantitative tissue characterization that does not require gadolinium-based contrast agents [13–15]. T2-mapping is an MRI technique that allows the detection of myocardial edema by reflecting increased water content in the heart muscle [16]. Native T1-mapping is sensitive to diffuse interstitial fibrosis and myocardial edema. Extracellular volume, which might be expanded in many cardiac pathologies, can be quantified using pre- and post-contrast T1-mapping measurements together with hematocrit [17]. By combining these parameters, acute myocarditis—typically associated with myocardial edema and prolonged T1 and T2 relaxation times—can be differentiated from myocardial fibrosis seen in patients with cardiomyopathies or previous myocarditis, which usually presents with elevated T1 values and normal T2 values [18, 19]. Acute graft rejection has similar patterns to acute myocarditis, as both involve inflammatory processes within the myocardium, leading to cardiomyocyte injury and changes in the extracellular matrix through increased vascular permeability, infiltration by inflammatory cells, and fluid accumulation in the interstitial space [20].

Quantitative cardiac imaging techniques for diagnosing myocarditis are already a routine part of clinical practice at leading cardiology centers worldwide [21]. However, the application of multiparametric MRI techniques to assess HTx rejection has not been sufficiently explored. Therefore, the aim of this study was to evaluate the use of quantitative cardiac MRI techniques, employing T1- and T2-mapping sequences, to assess graft tolerance and rejection within the 6 months following HTx.

## Patients and methods

The study was conducted in compliance with the Declaration of Helsinki, the ISHLT ethics statement, and was approved by the Wroclaw Medical University Ethical Committee (no. 687/2022, Wroclaw, 6 October 2022). All patients signed the written informed consent to participate in the study. The consent form was also approved by the Ethical Committee. All data were processed anonymously and with full confidentiality.

### Study population

This prospective, single-center study was performed between July 2023 and May 2024 at the Institute of Heart Diseases, University Hospital in Wroclaw, Poland. The study group included 17 patients aged 18 years or older who had undergone HTx within the preceding month. Exclusion criteria were as follows: severe claustrophobia precluding cardiac MRI; inability to remain in a supine position for at least 20 min or to follow breathing instructions; and standard contraindications to cardiac MRI, including, but not limited to, the presence of a ferromagnetic intracranial vascular clip or a metallic intraocular foreign body. The control group included 10 patients without structural heart disease who underwent cardiac MRI with T1 and T2 myocardial mapping sequences. This group served to establish reference values for myocardial relaxation times.

Patients with histopathologically confirmed graft rejection (ISHLT grade ≥ 3) were identified [4] and compared with those without rejection in terms of cardiac MRI-derived parameters, specifically myocardial relaxation times in T1- and T2-mapping sequences.

### Endomyocardial biopsies and laboratory parameters

Htx recipients were managed according to a standardized post-transplant care protocol at Jan Mikulicz-Radecki University Hospital in Wroclaw, Poland. The protocol included routine clinical assessments comprising medical history, physical examination, laboratory testing, echocardiography, and endomyocardial biopsies. Per protocol, biopsies are routinely performed at 1, 2, 3, 4, 6, and 8 weeks after transplantation, and at 3, 4, 6, 8, 10, 12, 18, and 24 months after transplantation. Biopsy results in our patients were correlated with myocardial relaxation times derived from cardiac MRI using T1- and T2-mapping sequences.

Myocardial biopsy samples were evaluated by an experienced cardiovascular pathologist according to ISHLT classification [4]. Grade 0 indicates no evidence of rejection changes; grade 1 indicates mild cellular rejection (1a, focal perivascular and/or interstitial infiltrate without myocyte damage; 1b, diffuse infiltrate without myocyte damage); grade 2 reflects moderate (focal) cellular rejection with a single focus of infiltrate and associated myocyte damage; grade 3 indicates moderate cellular rejection (3a, multifocal infiltrates with myocyte damage; 3b, diffuse infiltrate with myocyte damage); and grade 4 represents severe rejection characterized by diffuse inflammation with extensive myocyte damage, necrosis, hemorrhages, vasculitis, and aggressive lymphocytic and granulocytic infiltration.

Clinically significant acute cellular graft rejection (ACR), which typically requires intensification of immunosuppressive therapy and hospitalization, is defined as an ISHLT grade ≥ 3 (i.e., 3a, 3b, and 4). In cases of clinically significant ACR, patients were treated according to the institutional protocol. Therapy consisted of intravenous methylprednisolone administered for three consecutive days, followed by oral prednisone started at 1 mg/kg body weight with gradual tapering. Approximately 1 week after the ACR diagnosis, a control EMB was performed. When logistically feasible, an additional CMR examination was also carried out to reassess myocardial tissue characteristics after treatment.

Laboratory tests included the measurement of standard cardiac biomarkers: N-terminal pro–B-type natriuretic peptide (NT-proBNP) and troponin.

### Cardiac magnetic resonance imaging

Each patient underwent 5–6 cardiac MRI studies scheduled on the same day as the endomyocardial biopsy (± 1 day), including 4–5 noncontrast scans and one contrast-enhanced scan, typically performed as the final examination, unless there were contraindications such as patient refusal or severe chronic kidney disease with estimated glomerular filtration rate < 30 mL/min/1.73 m².

Cardiac MRI was performed during breath-holding at end-expiration using a 1.5-Tesla MRI scanner (Siemens Magnetom Sola Cardiovascular Edition, Siemens Healthineers). The imaging protocol included standard cine imaging using a balanced steady-state free precession sequence (short-axis stack covering the entire left ventricle and two-, three-, and four-chamber views) to assess myocardial morphology, volumes, and function; T2-weighted imaging; T1-mapping (3 short-axis views covering the basal, midventricular, and apical segments of the left ventricle); and T2-mapping (short-axis stack covering the entire left ventricle). Late gadolinium enhancement (LGE) imaging was performed to assess the presence of myocardial scar tissue using the same orientations as the cine images. A phase-sensitive inversion recovery sequence was acquired approximately 10–15 min after administration of gadobutrol (Bayer Inc.) at a dose of 0.15 mL/kg of body weight. LGE was visually assessed for its presence, pattern, and extent according to the AHA segmentation model. Regional subendocardial or transmural LGE was interpreted as ischemic in origin, whereas midmyocardial and subepicardial LGE were considered nonischemic [22].

Cardiac MRI images were analyzed using commercially available software. Volume quantification and ejection fraction measurements were performed with CVI42 (Circle Cardiovascular Imaging Inc.), while T1- and T2-mapping analyses were conducted using syngo.via (Siemens Healthineers). For T1- and T2-mapping, regions of interest were carefully drawn along the endocardial and epicardial borders of each myocardial segment, based on the American Heart Association (AHA) classification, in basal, midventricular, and apical short-axis slices. All cardiac MRI analyses were performed by an experienced reader.

### Statistical analysis

Statistical analysis was performed using Statistica (version 13.3; TIBCO Software Inc., StatSoft Polska Sp. z o.o.). Continuous variables were presented as mean ± standard deviation (SD). The assumption of normality was assessed for all studied parameters using the Shapiro–Wilk test. Variables with a skewed distribution were log-transformed to normalize their distributions. Categorical variables were presented as numbers and percentages.

Basic demographic and clinical parameters between HTx recipients with and without ACR were analyzed using the Mann–Whitney U test for continuous variables and the chi-square test for categorical variables. Differences in cardiac CMRI mapping parameters between the control group and study groups (without ACR and with ACR) were assessed using linear mixed-effects models. The association between cardiac MRI parameters, histopathological findings, and blood test results was assessed using a logistic generalized linear mixed model with a binomial distribution. Results are presented as odds ratios (OR) with 95% confidence intervals (95% CI) and *p*-values. The optimal cutoff value for T2-mapping to predict ACR was determined using the receiver operating characteristic (ROC) curve analysis. A *p*-value of 0.05 or lower was considered significant.

## Results

### Patient characteristics

From June 2023 to February 2024, 17 patients who underwent HTx at our institution met the study inclusion criteria. The first cardiac MRI examinations in this group were performed 19 to 29 days after transplantation (mean: 24 ± 4 days), once patients were clinically stable to be discharged from the postoperative intensive care unit and transferred to the regular heart transplant unit. Fifteen patients underwent 5 consecutive cardiac MRI studies, and 2 patients underwent 6 studies, yielding a total of 87 cardiac MRI examinations, each with a corresponding histopathological result from an endomyocardial biopsy. The final cardiac MRI studies were performed between 81 and 188 days (mean: 137 ± 32 days) after transplantation.

The study group included 15 men (88%) and 2 women (mean age: 53 ± 13 years; range: 29–69 years). The most common etiology of heart failure before HTx was nonischemic cardiomyopathy (71%). The mean age of heart donors was 40 ± 10 years (range: 19–54 years). The mean time of cold ischemia was 126 ± 49 min.

During the study period, 9 patients (53%) experienced ACR, comprising a total of 13 episodes (15% of the 87 endomyocardial biopsies). These episodes were classified as clinically significant cellular rejection endpoints. The mean time to ACR in the studied population was 84 ± 37 days, and the first studied episode of ACR occurred in patient 25 days after HTx. In 7 ACR episodes (in 6 patients), patients underwent an additional follow-up CMR together with a control endomyocardial biopsy approximately 1 week after treatment.

There were no differences in basic demographic and clinical parameters between Htx recipients with and without ACR (Table 1).

**Table 1:** Comparison of basic demographic and clinical data in heart transplant recipients without and with acute cellular rejection

Demographic and clinical data	Patients without acute cellular rejection *n* = 8	Patients with acute cellular rejection *n* = 9	*p*-value
Age, years	53 ± 13	53 ± 13	1.00
Men, *n* (%)	8 (100)	7 (78)	0.16
Body mass index, kg/m^2^	25.8 ± 3.3	24.9 ± 3.9	0.85
Nonischemic cardiomyopathy, *n* (%)	6 (75)	6 (67)	0.71
Age of heart donors, years	42 ± 9	38 ± 12	0.53
Time of cold ischemia, minutes	129 ± 53	123 ± 49	0.60

Results are presented as the number of patients (and percentage) and mean ± standard deviation

### Late gadolinium enhancement

LGE imaging was available in 14 patients, with enhancement present in 7 (50%). In all cases, the pattern was nonischemic, showing subepicardial or midmyocardial distribution. The extent of LGE was limited, involving a mean of 2.1 ± 0.9 myocardial segments.

### T1- and T2-mapping: a comparison with the control group

**Fig. 1:**
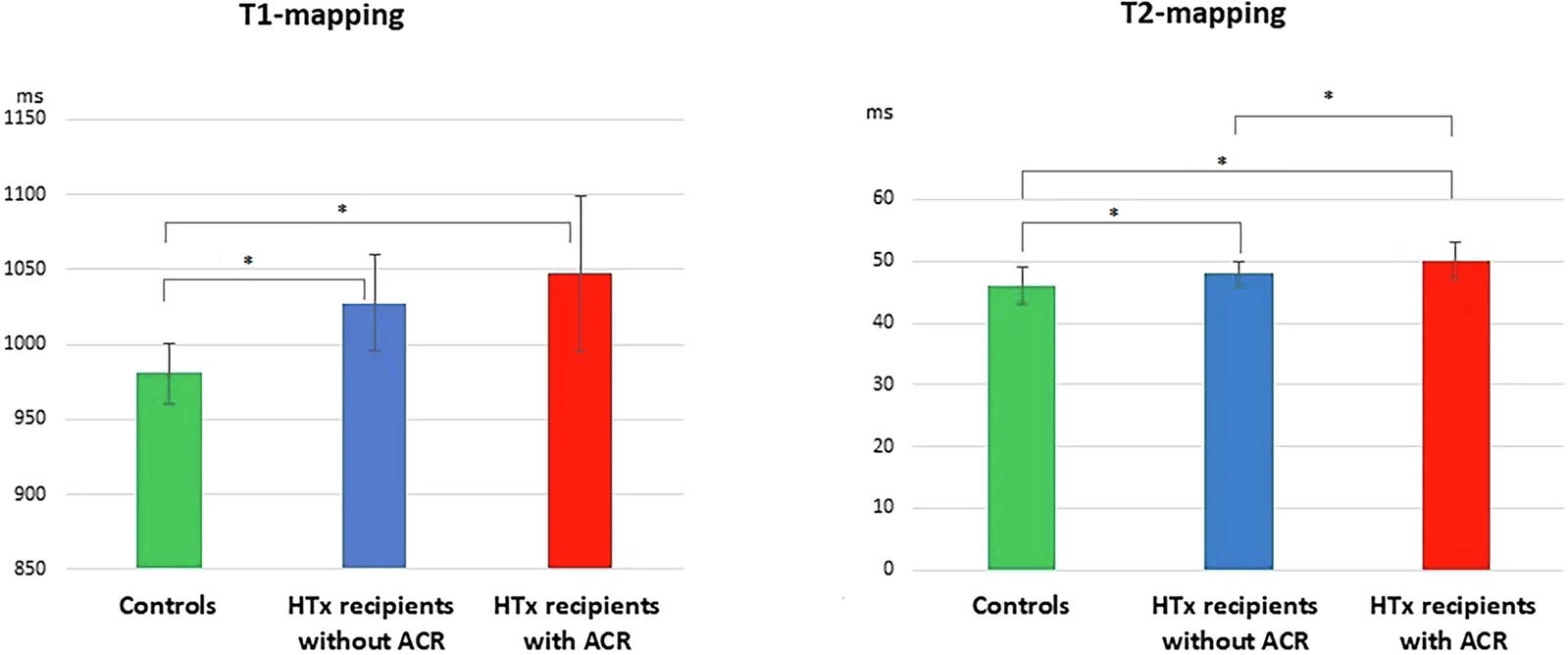
Comparison of mean T1- and T2-mapping values in heart transplant recipients with and without acute cellular rejection and in controls. * A *p*-value of 0.05 or lower was considered significant

To determine reference values for T1- and T2-mapping, all myocardial segments of the left ventricle in each control subject were analyzed, and the average values were included in further analysis. Significant differences were noted between controls (without cardiovascular disease in their medical history) and HTx recipients without ACR, both in T1-mapping (981 ± 20 vs. 1027 ± 35 ms, *p* = 0.001) and T2-mapping (46 ± 3 vs. 48 ± 2 ms, *p* = 0.007). For patients with ACR, the mean values were 1047 ± 52 ms for T1-mapping and 50 ± 3 ms for T2-mapping, both showing significant differences when compared with controls (both *p* < 0.001). Visual comparison of mean T1- and T2-mapping values in heart transplant recipients with and without ACR and in controls is presented in Fig. 1.

In daily clinical practice, a simplified method is used for quick assessment of T1- and T2-mapping, based on values from the midventricular inferoseptal segment of the left ventricle. Using this approach, a significant difference in T1- and T2-mapping values was observed between controls and HTx recipients without ACR (982 ± 16 vs. 1051 ± 46 ms, *p* = 0.002 and 45 ± 2 vs. 49 ± 4 ms, *p* = 0.009). In the ACR group, mean T1 and T2 values in the midventricular inferoseptal segment were 1085 ± 82 ms and 52 ± 4 ms, respectively, both significantly higher than in the control group (both *p* < 0.001).

### Comparison of laboratory and cardiac MRI parameters by acute cellular rejection status

No differences in the levels of NT-proBNP (1024 [570; 2557] vs. 2040 [736; 2975] pg/mL, *p* = 0.82) and troponin (17 [7; 36] vs. 17 [11; 52] pg/mL, *p* = 0.10; abnormal troponin measurements: 54 vs. 53%, *p* = 0.94) were noted between HTx recipients with and without ACR. The results of parametric cardiac MRI parameters, including T1- and T2-mapping values by myocardial segment (based on the AHA classification of left ventricular segments), stratified by the presence or absence of histopathological cellular rejection in endomyocardial biopsies, are presented in Tables 1 and 2.

**Table 2:** T1-mapping values (ms) in myocardial segments of heart transplant recipients with or without acute cellular rejection in endomyocardial biopsies

Myocardial segment	No cellular rejection *n* = 74	Cellular rejection *n* = 13	OR	95% CI	*p*-value
Basal anterior segment (1 AHA)	1017 ± 37	1045 ± 33	1.02	1.01–1.04	0.01*
Basal anteroseptal segment (2 AHA)	1052 ± 47	1067 ± 55	1.01	0.99–1.02	0.32
Basal inferoseptal segment (3 AHA)	1064 ± 45	1092 ± 60	1.01	1.00–1.03	0.04*
Basal inferior segment (4 AHA)	1044 ± 59	1055 ± 60	1.00	0.99–1.02	0.39
Basal inferolateral segment (5 AHA)	1022 ± 49	1038 ± 56	1.01	0.99–1.02	0.26
Basal anterolateral segment (6 AHA)	1010 ± 34	1046 ± 56	1.02	1.01–1.04	0.003 *
All basal segments	1035 ± 35	1057 ± 45	1.02	1.00–1.03	0.03*
Mid anterior segment (7 AHA)	1011 ± 36	1032 ± 48	1.01	1.00–1.03	0.08
Mid anteroseptal segment (8 AHA)	1027 ± 45	1043 ± 58	1.01	1.00–1.02	0.21
Mid inferoseptal segment (9 AHA)	1051 ± 46	1085 ± 82	1.01	1.00–1.02	0.04*
Mid inferior segment (10 AHA)	1033 ± 48	1057 ± 78	1.01	1.00–1.02	0.12
Mid inferolateral segment (11 AHA)	1007 ± 46	1024 ± 68	1.01	1.00–1.02	0.24
Mid anterolateral segment (12 AHA)	1009 ± 43	1031 ± 37	1.01	1.00–1.03	0.09
All midventricular segments	1023 ± 34	1045 ± 54	1.01	1.00–1.03	0.053
Apical anterior segment (13 AHA)	1011 ± 35	1023 ± 62	1.01	0.99–1.02	0.40
Apical septal segment (14 AHA)	1025 ± 46	1058 ± 77	1.01	1.00–1.02	0.04
Apical inferior segment (15 AHA)	1037 ± 56	1042 ± 84	1.00	0.99–1.01	0.79
Apical lateral segment (16 AHA)	1022 ± 50	1036 ± 68	1.00	0.99–1.02	0.40
All apical segments	1024 ± 35	1040 ± 61	1.01	1.00–1.02	0.18
Whole myocardium	1027 ± 35	1047 ± 52	1.02	1.00–1.03	0.06

Results are presented as mean and standard deviation of T1-mapping (ms)

*AHA* American Heart Association (cardiac segment model), *CI* confidence intervals, *OR* odds ratios

* A *p*-value of 0.05 or lower was considered significant

**Fig. 2:**
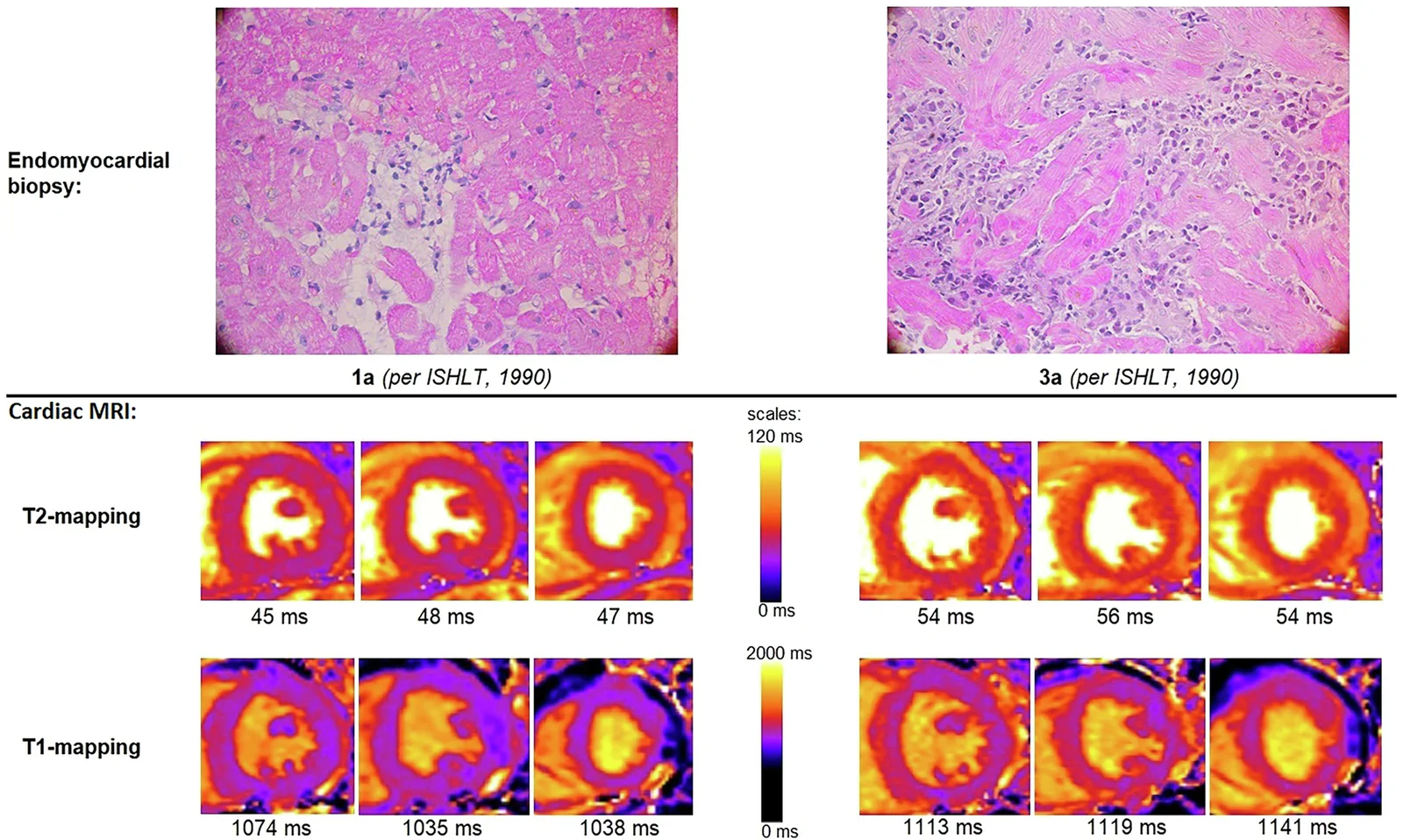
Microscopic view of endomyocardial biopsy samples presented alongside T2- and T1-mapping findings from cardiac magnetic resonance imaging in a representative patient after heart transplantation with an acute cellular rejection. The top panel presents the results of two endomyocardial biopsies from a 39-year-old male heart transplant (HTx) recipient. The figure on the left shows a biopsy performed 6 weeks after HTx, revealing grade 1a acute cellular rejection (clinically nonsignificant, according to ISHLT 1990): a scant perivascular lymphocytic infiltrate without significant degenerative changes in cardiomyocytes. The image on the right shows a biopsy performed 4 months post-HTx demonstrating grade 3a acute cellular rejection (clinically significant, according to ISHLT 1990): diffuse interstitial lymphocytic infiltrations consist of enlarged, stimulated lymphocytes, an admixture of histiocytes, and single eosinophils, which significantly separate individual cardiomyocytes; interstitial edema, amorphous material (collagen), and contraction bands are also visible. Both images: × 200 magnification, hematoxylin and eosin staining. The lower panels show images in the cardiac magnetic resonance imaging (MRI) corresponding to the histopathological findings (shown in the top panel). T2-mapping and T1-mapping are displayed as color-coded maps of the left ventricular short-axis segments at the basal, midventricular, and apical levels (from left to right, respectively). The numbers below each cardiac MRI image indicate the mean T2- and T1-mapping values for respective segments. ISHLT, International Society of Heart-Lung Transplantation; MRI, magnetic resonance imaging

In T1-mapping, patients with ACR showed higher values in the basal anterior (AHA segment 1), basal inferoseptal (AHA segment 3), basal anterolateral (AHA segment 6), midventricular inferoseptal (AHA segment 9), and apical septal (AHA segment 14) regions of the left ventricle compared with those without ACR. However, no significant differences were observed in global myocardial T1 values (Table 2). In T2-mapping, patients with ACR had significantly higher global myocardial values than patients without ACR, as well as elevated values in the basal inferoseptal (AHA segment 3), basal anterolateral (AHA segment 6), midventricular septal (AHA segments 8 and 9), apical septal (AHA segment 14), and apical lateral (AHA segment 16) regions (Table 3). A visual example of an endomyocardial biopsy specimen presented alongside T2- and T1-mapping findings from cardiac MRI in an HTx recipient is shown in Fig. 2.

**Table 3:** T2-mapping values (ms) in myocardial segments of heart transplant recipients with and without acute cellular rejection in endomyocardial biopsies

Myocardial segment	No cellular rejection *n* = 74	Cellular rejection *n* = 13	OR	95% CI	*p*-value
Basal anterior segment (1 AHA)	48 ± 3	49 ± 4	1.16	0.96–1.39	0.12
Basal anteroseptal segment (2 AHA)	48 ± 3	49 ± 3	1.13	0.95–1.35	0.17
Basal inferoseptal segment (3 AHA)	48 ± 3	50 ± 4	1.33	1.08–1.64	0.01*
Basal inferior segment (4 AHA)	48 ± 4	49 ± 4	1.12	0.95–1.31	0.19
Basal inferolateral segment (5 AHA)	47 ± 3	49 ± 3	1.20	0.98–1.48	0.08
Basal anterolateral segment (6 AHA)	47 ± 3	49 ± 4	1.24	1.01–1.52	0.04*
All basal segments	48 ± 2	49 ± 3	1.30	1.03–1.65	0.03*
Mid anterior segment (7 AHA)	48 ± 3	49 ± 5	1.10	0.94–1.30	0.24
Mid anteroseptal segment (8 AHA)	49 ± 3	51 ± 5	1.22	1.03–1.45	0.02*
Mid inferoseptal segment (9 AHA)	49 ± 4	52 ± 4	1.20	1.03–1.39	0.02*
Mid inferior segment (10 AHA)	47 ± 4	48 ± 4	1.08	0.93–1.25	0.31
Mid inferolateral segment (11 AHA)	47 ± 3	49 ± 4	1.16	0.99–1.36	0.06
Mid anterolateral segment (12 AHA)	47 ± 3	48 ± 4	1.09	0.92–1.29	0.30
All midventricular segments	48 ± 3	50 ± 4	1.20	1.00–1.44	0.053
Apical anterior segment (13 AHA)	49 ± 3	51 ± 5	1.14	0.97–1.34	0.12
Apical septal segment (14 AHA)	49 ± 3	51 ± 5	1.18	1.02–1.36	0.02*
Apical inferior segment (15 AHA)	48 ± 3	49 ± 4	1.13	0.93–1.36	0.21
Apical lateral segment (16 AHA)	48 ± 3	50 ± 3	1.22	1.01–1.48	0.04*
All apical segments	49 ± 3	50 ± 4	1.27	1.03–1.57	0.02*
Whole myocardium	48 ± 2	50 ± 3	1.31	1.04–1.64	0.02*

Results are presented as mean and standard deviation of T2-mapping (ms)

*AHA* American Heart Association (cardiac segment model), *CI* confidence intervals, *OR* odds ratios

* A *p*-value of 0.05 or lower was considered significant

**Fig. 3:**
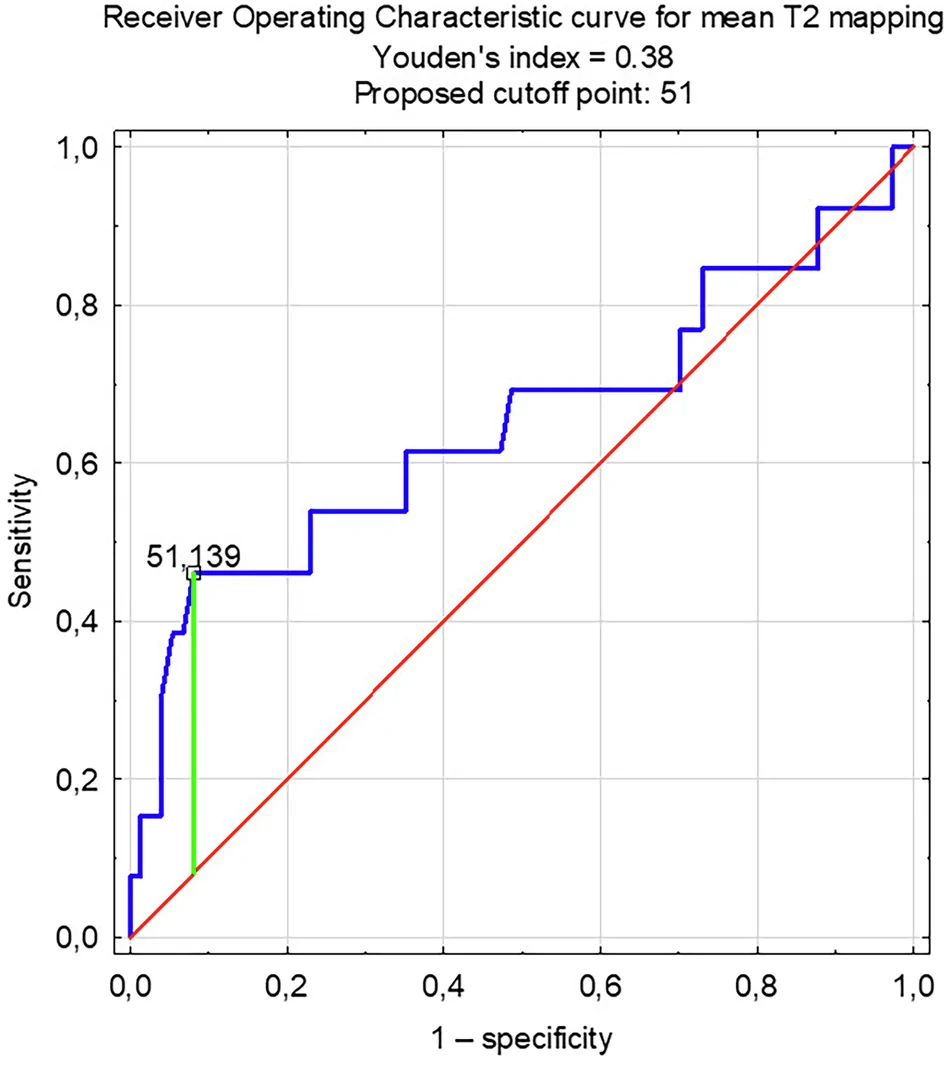
Receiver operating characteristic curve analysis for mean T2-mapping as a marker of acute cellular rejection

ROC analysis showed an area under the curve of 0.65 (95% CI: 0.46–0.84), the mean T2-mapping value in the identification of ACR. The optimal cutoff value of 51 ms yielded a sensitivity of 46% and a specificity of 92% (Fig. 3).

## Discussion

In this prospective single-center study, we investigated the potential utility of quantitative cardiac MRI techniques, specifically T1- and T2-mapping, in detecting ACR in HTx recipients within the first 6 months after surgery. Our findings suggest that the T2-mapping value may serve as a noninvasive imaging biomarker for transplant rejection, differentiating patients with and without histopathologically confirmed ACR. While T1-mapping revealed some regional differences, it was less consistent across the entire myocardium. Notably, standard cardiac biomarkers, troponin and NT-proBNP, did not differ significantly between patients with and without ACR, suggesting their limited utility for detecting rejection after HTx.

One of the key findings of our study is that T2 myocardial relaxation times were significantly elevated in patients with ACR compared with both HTx recipients without rejection and healthy controls. The diagnostic performance of T2-mapping, although modest in sensitivity, demonstrated high specificity for detecting significant ACR at an optimal cutoff value of 51 ms. Although the specificity was high, the sensitivity was relatively low, suggesting that T2-mapping may be more useful as a supportive imaging marker rather than a standalone screening tool for rejection. Future approaches combining T2-mapping with advanced image analysis techniques, such as radiomics or machine learning–based pattern recognition, may further improve noninvasive rejection detection. It is well established that myocardial edema—seen in acute cardiac conditions such as myocarditis or myocardial infarction—can be detected using T2-weighted imaging and the more modern sequence of T2-mapping [18, 23, 24]. This is possible because increased water content leads to prolongation of T2 relaxation times; thus, myocardial edema resulting from immune-cell infiltration and tissue injury can be readily detected on cardiac MRI [24]. Similarly, myocardial edema is present in ACR results from the infiltration of inflammatory cells and interstitial fluid accumulation, driven in part by increased vascular permeability [20]. Beyond its diagnostic role, T2-mapping may provide dynamic information on myocardial inflammatory activity and could potentially be used to monitor response to augmented immunosuppressive therapy after clinically significant ACR. Importantly, absolute T2 values and corresponding diagnostic thresholds depend on scanner type, field strength, and acquisition protocol. Therefore, the cutoff of 51 ms identified in our cohort reflects local acquisition conditions and should not be directly extrapolated to other centers without validation. These considerations highlight the need for multicenter standardization efforts and center-specific reference ranges when implementing T2-mapping for rejection surveillance.

Relatively few cardiac MRI studies have been conducted in patients after HTx, and those that exist often involve small study populations and use different definitions of significant allograft rejection, making it difficult to standardize recommendations. One of the first pilot studies in this patient population was published in 2014 by Miller et al, but it failed to demonstrate significant differences in multiparametric cardiac MRI findings between patients with and without ACR during the early post-transplantation period [25]. However, more recent studies indicated the potential utility of cardiac MRI in the assessment of allograft rejection. Notably, Anthony et al presented key findings supporting this approach in a prospective, randomized, single-center, noninferiority pilot study conducted from 2018 to 2020. In this study, 40 HTx recipients were randomized 4 weeks after surgery into two groups based on the method of ACR monitoring: (1) standard protocol with endomyocardial biopsies and (2) a cardiac MRI-based multiparametric mapping approach, including T1- and T2-mappings [13]. The results demonstrated that multiparametric cardiac MRI could accurately detect significant cardiac allograft rejection, guide immunosuppressive therapy, and reduce the number of invasive biopsy procedures during the first year after HTx [13]. The diagnostic performance observed in our study was lower than that reported by Anthony et al, who found AUC values of 0.897 for T1-mapping and 0.938 for T2-mapping. Several methodological differences between the studies may explain this discrepancy. First, our study used standard breath-hold clinical sequences on a routine 1.5-T scanner, reflecting routine clinical practice, whereas Anthony et al applied a respiratory-navigated motion-corrected sequence for T2-mapping that may improve measurement precision. Second, we analyzed relaxation times across the entire left ventricular myocardium, while their measurements were focused on midventricular regions, particularly the septum, which typically provide more stable values. Third, differences in the reference standard may also have contributed. In the study by Anthony et al, endomyocardial biopsy samples were evaluated by experienced transplant pathologists with independent assessment and consensus adjudication, whereas in our study, biopsies were assessed as part of routine clinical practice by a single experienced histopathologist. Interobserver variability in the histopathological assessment of endomyocardial biopsies is well recognized and may influence estimates of diagnostic performance [26]. Finally, differences in study endpoints, as well as the smaller sample size in our cohort, may also have influenced the observed diagnostic performance. Taken together, these differences suggest that the diagnostic performance of quantitative cardiac MRI parameters may depend on acquisition protocols, analytical strategies, and study design. Importantly, our study reflects routine clinical practice in a heart transplant center, where cardiac MRI acquisitions follow standard protocols and biopsy specimens are assessed according to routine histopathological practice, which may differ from highly controlled research settings.

In parallel with cardiac MRI-based approaches, other imaging modalities are being explored for the detection of cardiac allograft injury and guidance of clinical management. Cardiac computed tomography enables noninvasive visualization of the coronary arteries, and recent studies have demonstrated progressive coronary artery calcium accumulation and a decline in computed tomography–derived fractional flow reserve, reflecting the development of cardiac allograft vasculopathy during long-term follow-up [27, 28]. While computed tomography primarily provides anatomical and functional assessment of the coronary arteries [29], cardiac MRI offers detailed myocardial tissue characterization, highlighting the complementary roles of these imaging techniques.

In our study, T1-mapping demonstrated significant differences between patients with and without ACR only in selected myocardial segments. Interestingly, these abnormalities showed a consistent pattern involving the inferoseptal segments at basal and midventricular levels and the apical septal segment. This distribution may reflect the region most commonly sampled during routine endomyocardial biopsy. This may indicate that T1-mapping is more sensitive to diffuse or localized myocardial fibrosis and less specific for acute inflammatory changes. Our findings suggest that, while T1-mapping may contribute to tissue characterization in the transplant population, its utility in identifying acute rejection appears limited without corroborating evidence from T2-mapping or other imaging modalities. This is consistent with recent publications highlighting the need for a multiparametric mapping approach in the cardiac MRI-based diagnosis of allograft rejection [30, 31].

The main limitation of this study is the small cohort of HTx recipients; all treated at a single center and examined using a scanner from a single vendor. However, the number of evaluated events was increased through multiple sequential examinations in each patient, yielding a satisfactory number of endpoints. As this is an exploratory study including a limited number of patients, the estimates of effect size and diagnostic performance (ROC/AUC) should be interpreted with caution and require validation in larger cohorts. Therefore, there is an urgent need for further studies—ideally, prospective, multicenter, randomized trials—to assess the true effectiveness of cardiac MRI in detecting cellular rejection across different scanner vendors and magnetic field strengths (1.5 and 3.0 Tesla).

Our study highlights that T2-mapping may serve as a supportive noninvasive imaging biomarker for identifying patients with a higher likelihood of clinically significant ACR in Htx recipients, particularly within the first 6 months post-surgery. Importantly, in the setting of heart transplantation, noninvasive imaging techniques are typically applied within structured rejection surveillance programs in a high-risk population rather than as population-based screening tools, and imaging markers with high specificity may therefore still provide clinically meaningful information for risk stratification and guiding further diagnostic evaluation. Conversely, T1-mapping revealed limited diagnostic utility, showing regional differences but lacking consistency across the myocardium. Its role may be more suited to detecting fibrosis rather than acute inflammatory changes. Therefore, multiparametric cardiac MRI approaches, combining T1- and T2-mapping, may enhance diagnostic accuracy and support more targeted use of invasive endomyocardial biopsies. However, further studies in larger and preferably multicenter cohorts are warranted to better define the clinical role of these techniques across different imaging platforms and patient populations.

## Conclusions

Noninvasive, quantitative cardiac MRI techniques may help assess the risk of heart transplant rejection and complement current biopsy-based surveillance in Htx recipients. The usefulness of this approach in clinical practice needs further study.

## Abbreviations

ACR: Acute cellular rejection
AHA: American Heart Association
CI: Confidence interval
HTx: Heart transplantation
LGE: Late gadolinium enhancement
MRI: Magnetic resonance imaging
NT-proBNP: N-terminal pro–B-type natriuretic peptide
OR: Odds ratios
ROC: Receiver operating characteristic
